# Experimental Study on the Physical Properties and Freeze–Thaw Resistance of Rice Straw–Mortar Composites

**DOI:** 10.3390/ma19143020

**Published:** 2026-07-13

**Authors:** Rongfei Zhao, Jiajun Hou, Binbin Feng, Xinyan Yi, Huaqian Zhang, Wei Gao

**Affiliations:** 1 College of Water Conservancy, Shenyang Agricultural University, Shenyang 110866, China; rongfeizhao@syau.edu.cn (R.Z.); 2024240188@stu.syau.edu.cn (J.H.); fff1322026@163.com (X.Y.); r13455@126.com (H.Z.); 2 College of Engineering, Shenyang Agricultural University, Shenyang 110866, China

**Keywords:** physical properties, rice straw-mortar composite, optimal mix ratio, freeze-thaw cycling test

## Abstract

With the increasing demand for the resource utilization of agricultural waste and the development of sustainable building materials, rice straw–mortar composites have attracted considerable attention owing to their renewability and environmental friendliness. In this study, a three-factor and three-level regression analysis experiment was carried out using Design-Expert software to obtain the optimal physical properties and mix ratio of rice straw–mortar composite materials. With an optimal mix ratio of 15% gypsum, 20 mm straw length, and 15% straw content, the composite achieved a compressive strength of 3.06 MPa, a dry density of 1.340 g·cm^−3^, and a water absorption of 20.8%. A freeze–thaw cycling test of 15 cycles from −20 °C to 20 °C resulted in a strength loss of 32.2–47.2%, a mass loss of 2.1–4.5%, and water absorption of 21.8–37.2%. The results suggest that rice straw–mortar composites may be suitable for non-load-bearing wall applications under freeze–thaw exposure conditions.

## 1. Introduction

China is one of the world’s largest producers of crop straw, with its annual crop straw output reaching 0.97 × 10^9^ tons [1]. Being a common agricultural practice in China, open crop straw burning represents an important source of atmospheric pollutants [2]. Open-field crop residue burning has been reported to cause significant adverse health effects, including coughing, eye irritation, headaches, and respiratory symptoms [3]. Therefore, people have made comprehensive use of straw resources, and the main utilization methods are feed, base material, energy, fertilizer, and raw material [4,5].

In recent years, straw has been increasingly incorporated into cement-based materials due to its low cost, renewability, and favorable physical properties. Niu et al. [6] investigated the application of corn straw in cement-based composites and demonstrated the feasibility of utilizing agricultural straw in cementitious materials, providing a promising approach for the development of sustainable building materials. Hasan et al. [7] studied concrete incorporating corn cob ash and corn straw fibers, and found that an optimal fiber content improved the interfacial bonding and significantly enhanced the mechanical properties of the material. Petrella et al. [8] conducted a series of tests to evaluate the mechanical properties of cement mortars incorporating wheat straw and to investigate the pore structure of both the straw and the interfacial transition zone between the straw and the cement matrix. The results demonstrated that straw-modified mortars are suitable for lightweight construction applications. Chen et al. [9] demonstrated that the incorporation of natural fibers into concrete can inhibit crack propagation, enhance tensile strength, and improve the stress distribution within the matrix, thereby enhancing the mechanical performance of the material. However, despite its numerous advantages, the incorporation of straw into cement-based materials can still introduce certain adverse effects. Zhang et al. [10] found that the compressive and flexural strengths of concrete incorporating natural rod-shaped straw decrease significantly with the increase in straw content. Hamada et al. [11] pointed out that, although natural fibers can improve certain mechanical properties of cement-based materials, their high water absorption and weak fiber–matrix interfacial bonding may adversely affect the overall performance and durability of the composites. Liu et al. [12] found that increasing the straw content led to a reduction in the strength of cement mortar.

It is noteworthy that the performance of straw mortar concrete is closely related to the composition of the material, with straw content, straw length, and gypsum dosage all influencing its properties. Zhao et al. [13] reported that straw content was negatively correlated with the compressive strength and dry density of straw mortar composites and positively correlated with water absorption. Doostkami et al. [14] found that an increase in rice straw fiber content led to a reduction in the compressive strength of concrete. He et al. [15] found that the compressive strength of concrete increased slightly when the corn straw fiber content was 0.8%, whereas a further increase in fiber content resulted in a reduction in compressive strength. Mechanistically, soluble sugars and organic acids leached from rice straw can inhibit early cement hydration, leading to a looser microstructure of hydration products and a reduction in matrix strength [16]. An increase in rice straw content further raises the concentration of these inhibitory substances, thereby intensifying the retardation of the hydration process. In addition to straw content, straw length also influences the performance of straw mortar concrete. Ding et al. [17] pointed out that variations in straw fiber length affect the dispersion of fibers within the cement matrix, which in turn influences the compactness and the physico-mechanical properties of straw–cement composites. Cao et al. [18] reported that the length of straw fibers significantly affects the strength and deformation behavior of cement-based materials, and that an optimal fiber length exists to achieve the best reinforcing effect. These effects can be attributed to the fact that fiber length regulates the compressive strength by influencing its dispersion uniformity within the mortar and the interfacial bonding with the matrix. Specifically, excessively long fibers tend to entangle with each other and result in non-uniform mixing, leading to insufficient wrapping by cementitious materials and the formation of voids at the interface, thereby reducing the strength, whereas fibers of moderate length can be uniformly dispersed and form good mechanical interlocking with the matrix, resulting in a slight improvement in compressive strength [19]. In addition to straw content and straw length, gypsum content is another important factor influencing the performance of straw mortar concrete. Jin et al. [20] reported that gypsum dosage had a significant influence on the compressive and flexural strengths of cement-based materials, and that an optimum gypsum content could be identified under specific curing conditions. Yan et al. [21] found that increasing gypsum content improved the microstructure and strength of cement-based composites up to an optimum level, whereas excessive gypsum led to increased porosity and a decline in strength. These effects can be attributed to the fact that gypsum regulates the formation of ettringite during the hydration process. The filling, cementation, and volumetric expansion effects of ettringite contribute to pore refinement and microstructural densification, thereby enhancing the mechanical properties of the material [22].

In practical engineering applications, mortar concrete is inevitably exposed to complex environmental conditions, among which freeze–thaw action is one of the most critical factors affecting its long-term performance and durability. Zhao and Lian [23] found that repeated freeze–thaw cycles led to the deterioration of the internal structure of concrete, accompanied by an increase in porosity and a reduction in compressive strength and elastic modulus. Lv et al. [24] reported that freeze-thaw cycles cause progressive deterioration of concrete properties, as evidenced by increased mass loss, decreased compressive strength, and reduced ultrasonic pulse velocity. Zhou et al. [25] found that the compressive strength and relative dynamic elastic modulus of high-strength concrete gradually decreased with an increasing number of freeze-thaw cycles. These effects can be attributed to the fact that during freeze–thaw cycles, the repeated freezing and thawing of pore water generates frost-heaving and hydraulic pressures, which induce the initiation and propagation of microcracks, deteriorate the pore structure, and reduce the compactness of the material, ultimately resulting in the degradation of its mechanical properties and durability [26]. Therefore, investigating the freeze-thaw resistance of straw-reinforced mortar concrete is essential for evaluating its long-term durability and ensuring its safe application in engineering projects in cold regions.

Previous studies have mainly focused on the individual effects of straw content, straw length, or binder content, while limited attention has been given to their combined and interactive influences on composite performance. In this study, the physical and mechanical properties of rice straw-mortar composite materials, such as, compressive strength, dry density and water absorption rate are improved by adjusting the influencing factors such as gypsum content, straw length and straw content. Meanwhile, winter temperature in north of China is relatively low, and the average temperature of most area is as low as −20 °C. Because the composite materials contain considerable amounts of liquid water and entrapped air, repeated freeze-thaw action will lead to strength loss and structural damage of straw composite materials. Therefore, it is meaningful to study the action rules of various influencing factors on the physical and mechanical properties indexes of rice straw-mortar composites, achieve optimal values of the response variables under the corresponding optimal factor levels, and analyze the freeze–thaw cycles action mechanism of the composite materials from a macro perspective for the application and promotion of straw-mortar composite materials as building wall blocks in north of China.

## 2. Materials and Methods

### 2.1. Experimental Materials

#### 2.1.1. Portland Cement

Portland cement is one of the important raw materials for preparing rice straw mortar composite materials. It plays a major role in the mechanical strength of the composite materials and helps to enhance the stability of the test blocks. The cement used in this experiment was ordinary Portland cement produced by Shenyang Sunnsy Gongyuan Cement Co., Ltd (Shenyang, China). The physical and mechanical properties of cement are shown in Table 1.

#### 2.1.2. Gypsum

Yao [27] found that when the mass ratio of gypsum to cement was 1:9, the prepared mortar specimens exhibited a relatively high density, and their compressive strength remained within a relatively high range. The horizontal indicators for the gypsum content (based on the mass of cement) were determined to be 5%, 10%, and 15%.

The test used gypsum produced by Liaoning Sanling San Decoration Materials Co., Ltd., (Shenyang, China) which was in the form of white powder. The technical indicators of the gypsum are shown in Table 2.

#### 2.1.3. Rice Straw

Benmansour et al. [28] found that although the compressive strength of the composite decreases significantly compared to the plain matrix when the date palm fiber content ranges from 5% to 15%, both the mechanical strength and thermal conductivity still satisfy the performance requirements for lightweight concrete used as structural and insulating material. Feng [29] obtained that the straw cement composite mortar had better performance when the wheat straw content was 10–20%. Therefore, the rice straw content (the mass ratio of rice straw to cement) was selected as 15%, 20% and 25% in this study. Petrella et al. [30] found that, among wheat straw fibres of 4–6 mm, 13–17 mm, 34–37 mm, and 58–62 mm, longer straw fibres led to a corresponding increase in the strength of the cement-based composites. Wang et al. [31] studied corn straw fibers with lengths of 5 mm, 10 mm, and 15 mm; at a fiber content of 1%, the optimal length for mechanical strength was 10 mm, and the optimal length for crack resistance was 15 mm. In this study, three test levels of rice straw length were selected as 10, 15 and 20 mm.

The straw used in this experiment was from the rice plants of Shenyang Agricultural University. From the root to the ear, it was approximately 110 cm in total. After sun-drying, the material was adjusted to a moisture content of no more than 10%, the undecayed and undamaged rice straw was selected and the excess roots and ears were removed. It was then manually cut into strips of 10 ± 2 mm, 15 ± 2 mm, and 20 ± 2 mm in size, and stored in moisture-proof plastic bags. The major chemical constituents of rice straw are 35.6% cellulose, 20.5% hemicellulose, 16.48% lignin, 15.2% ash, and 12.1% moisture.

#### 2.1.4. Sand

The fine aggregate used in the experiment was natural river sand, which was taken from the Hun River in Liaoning Province. The fine aggregate had a fineness modulus of 2.5, an average particle size of 0.45 mm, and a clay content of 3.0%.

#### 2.1.5. Water

The experimental water was ordinary tap water.

### 2.2. Experimental Methods

#### 2.2.1. Preparation of Test Blocks

This experiment adopts a mixing process of dry mixing first and then wet mixing. The weighed cement, rice straw, sand, and gypsum are added to the JJ-5 cement mortar mixer (Wuxi Building Materials Testing Instrument Factory, Wuxi, China). After 3 min of dry mixing, water was gradually added, and mixing was continued for at least 5 min until a cohesive paste was formed. The mixer was then turned off. The surface of the 70.7 mm × 70.7 mm × 70.7 mm mortar triple mold is wiped clean. At the same time, a thin layer of mineral oil is evenly applied to the inner wall, and the mixed mortar is injected into it. The test blocks must be left to stand in a ventilated and dry indoor environment for 48 h, then the molds are removed, numbered, and placed 10–20 mm apart on the SJ-40A standard mortar curing chamber (Shenyang Zhongyan Testing Instrument Co., Ltd., Shenyang, China) set at a temperature of 20 ± 5 °C and a relative humidity of over 95% for 28 days of curing. The SJ-40A standard mortar curing chamber had a temperature control accuracy of ±2 °C and a relative humidity control accuracy of ±3%.

#### 2.2.2. Determination of Physical Properties

(1) Determination of compressive strength

**Figure 1:**
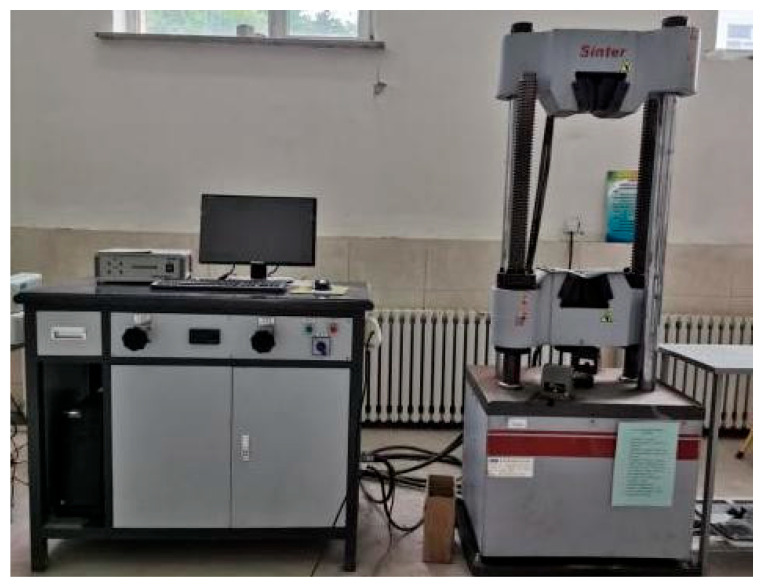
Universal testing machine.

The design specifications for compressive strength are based on the cube compressive strength test method in JGJ/T70-2009 “Standard for test method of performance on building mortar” [32]. The compressive test blocks have a size of 70.7 mm × 70.7 mm × 70.7 mm, and the average value of the strengths of 3 test blocks is taken. A WAW-300B microcomputer-controlled electro-hydraulic servo universal testing machine (Jinan Zhongluchang Testing Machine Manufacturing Co., Ltd., Jinan, China) was used. The testing machine had a maximum loading capacity of 300 kN. The compressive strength testing instrument is shown in the Figure 1. The compressive strength value of the test block is calculated according to Formula (1).

The experimental procedure was as follows:

(1) After confirming that the specimen was free from visible damage, it was placed on the lower platen of the testing machine with one side surface serving as the loading surface. The center of the specimen was carefully aligned with the center of the lower platen. The upper platen was then positioned on the top surface of the specimen, ensuring that its center was aligned with the center of the specimen.
(2) The testing machine was then started, and the specimen was subjected to a continuously and uniformly applied compressive load. The load was applied at a constant loading rate of 0.25 kN/s until specimen failure.
(3) When the specimen approached failure and exhibited visible deformation, no further adjustments were made to the loading rate. Loading was continued until the specimen failed, and the maximum failure load was recorded.
(4) The compressive strength of the specimen was calculated according to Formula (1) and reported to the nearest 0.01 MPa.

(1) $$f_{m,\mathrm{cu}}=1.35\frac{N_{u}}{A}$$

where: $f_{m,\mathrm{cu}}$ is compressive strength of testing block, MPa;

$N_{u}$ is failure load of testing block, N;

A is pressure bearing area of failure load of testing block, mm^2^.

(2) Determination of dry density

After the specimen with dimensions of 70.7 mm × 70.7 mm × 70.7 mm was protected for 28 days, it was placed in a 101-2 electric blast drying oven (Tianjin Shude Testing Instrument Co., Ltd., Tianjin, China). The oven operated at a rated voltage of 220 V and a frequency of 50 Hz, with a blower power of 30 W and a heating power of 2.5 kW. Refer to GB/T 20473—2006 “Dry-mixed thermal insulating composition for buildings” [33]. The constant temperature drying oven was slowly heated to 60 ± 5 °C. Every 3 h, the specimen was taken out for observation and data recording. This process was repeated until the mass of the specimen reached a constant value, and then it was moved to the room for cooling. Under constant temperature conditions, if the mass change rate of the specimen within 3 h was less than 0.2%, it was considered to have reached the constant mass $M_{0}$. The dry density was calculated according to Formula (2).

(2) $$\rho_{0}=\frac{M_{0}}{V}$$

where: $\rho_{0}$ is dry density of the test block, g·cm^−3^;

$M_{0}$ is the constant mass of the test block after it has been dried, g;

V is the volume of the test block, cm^3^.

(3) Determination of water absorption rate

After 28 days of curing, the specimens were immersed in water for 2 days and then removed. The mass $M_{1}$ of the specimens was determined using a YP802N electronic balance (Guangzhou Yongcheng Experimental Instrument Co., Ltd., Guangzhou, China). The balance had a maximum weighing capacity of 800 g and a readability of 0.01 g. Place the test block in a constant temperature drying oven maintained at a temperature of 60 ± 5 °C. The specimen was periodically removed and weighed at 3 h intervals until its mass reached a constant value $M_{0}$, and then transferred to room temperature for cooling. The water absorption rate of testing block was calculated according to Formula (3).

(3) $$W_{x}=\frac{M_{1}-M_{0}}{M_{0}}\times 100\%$$

where: $W_{x}$ is the water absorption rate of the test block, %;

$M_{1}$ is the mass of the test piece after being soaked in water for 2 days, g;

$M_{0}$ is the constant mass of the test block after it has been dried, g.

#### 2.2.3. Determination of Freeze-Thaw Resistance Performance

**Figure 2:**
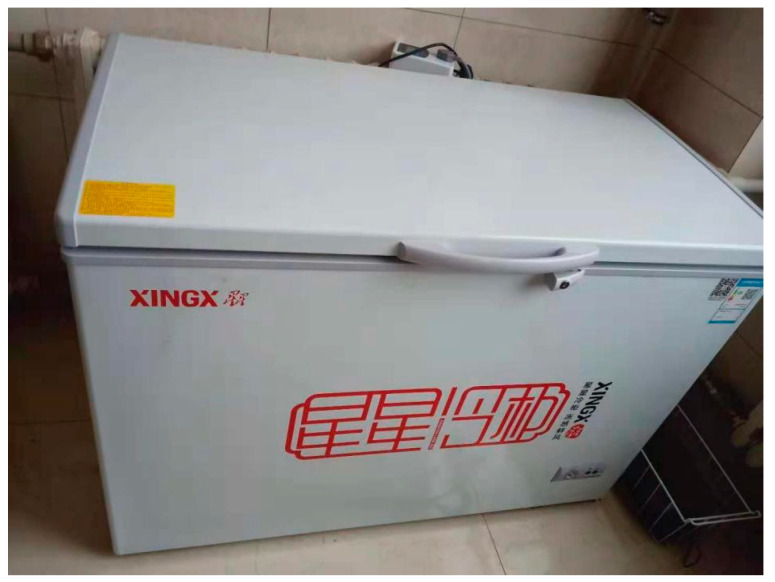
Test device of freeze-thaw cycles.

Antifreeze property refers to the performance of a material that can withstand repeated freeze-thaw cycles without being damaged when it is in a water-absorbed saturated state. Among them, the mass loss rate and strength loss rate are two important indicators for testing the freeze-thaw performance of test blocks. The freeze–thaw cycles were conducted using a BC-305E variable-temperature refrigerator-freezer (Zhejiang Xingxing Cold Chain Integration Co., Ltd., Taizhou, Zhejiang, China). The freezing and thawing device is shown in the Figure 2.

(1) The mass loss rate after freeze-thaw cycles is calculated by Formula (4).

(4) $$\Delta m_{m}=\frac{m_{0}-m_{n}}{m_{0}}\times 100\%$$

where: $\Delta m_{m}$ is the rate of mass loss after n cycles of freezing and thawing, %;

$m_{0}$ is the mass of the test piece before the freeze-thaw cycle test, g;

$m_{n}$ is the drying quality of the test block after n freeze-thaw cycles, g.

(2) The strength loss rate after freeze-thaw cycles is calculated by Formula (5).

(5) $$\Delta f_{m}=\frac{f_{m1}-f_{mn}}{f_{m1}}\times 100\%$$

where: $\Delta f_{m}$ is the rate of strength loss after n freeze-thaw cycles, g;

$f_{m1}$ is the average compressive strength value of the comparison test block, MPa;

$f_{\mathrm{mn}}$ is the compressive strength value after n freeze-thaw cycles, MPa.

### 2.3. Experimental Design

In this study, Design-Expert 13 software was used to design 17 sets of three-factor and three-levelBox-Behnken central combination experiments, which were carried out at room temperature with different levels of influencing factors. Main influencing factors and coded factor levels of independent variables are given in Table 3. The influence of various factors on the compressive strength, dry density and water absorption rate were investigated. The optimal values of the physical and mechanical properties of the rice straw–mortar composites and the corresponding factor levels were obtained.

Meanwhile, freeze–thaw cycle tests were conducted to determine the mass and compressive strength of the specimens after 5, 10, and 15 cycles. The mass loss rate and compressive strength loss rate were then calculated by comparing the results before and after freeze–thaw cycling. Analyzing the influence of freeze-thaw cycles on water absorption rate of composite blocks, which was related to block’s compression failure.

## 3. Results and Discussion

The results of 17 experiments are shown in the Table 4. In the table, the compressive strength ranges from 0.55 MPa to 3.00 MPa. The density ranges from 0.921 g·cm^−3^ to 1.549 g·cm^−3^. The water absorption ranges from 18.2% to 36.3%. Residual analysis shows that the residuals of all three datasets are scattered around zero without clear systematic deviation. The first two datasets have relatively small fluctuations, indicating good fitting performance and reliable prediction accuracy. The third dataset shows a wider spread, but the distribution remains random, suggesting that the model still captures the overall trend of the data reasonably well.

### 3.1. Modeling and Analysis of Variance

Used Design-Expert software to perform regression analysis on the data of Table 5, and fitted regression equations with gypsum content, straw length, and straw content as independent variables, compressive strength, dry density and water absorption rate as dependent variables. $R_{\mathrm{adj}}^{2}$ is an important coefficient standard for checking the reliability and accuracy of the regression model. The value is closer to 1, and the model fits better.

Variance analysis results of the regression equations in Table 5 are shown in Table 6.

In the optimization of compressive strength, the linear terms of gypsum content, straw length and straw content, as well as the quadratic terms of gypsum content, straw length and straw content, all exhibited highly significant effects. Among the interaction terms, the interaction between gypsum content and straw content, and the interaction between straw length and straw content, showed significant effects.

In the optimization of dry density, the linear term of straw content, the quadratic term of gypsum content and the quadratic term of straw content all had highly significant effects; the linear term of gypsum content and the linear term of straw length had significant effects on dry density.

In the optimization of water absorption, the linear term of straw content and the quadratic term of straw length showed highly significant effects. The linear terms of gypsum content and straw length, the interaction term between straw length and straw content, as well as the quadratic terms of gypsum content and straw content, all exhibited significant effects.

### 3.2. Influence of Interaction of Various Factors on the Physical and Mechanical Properties

**Figure 3:**
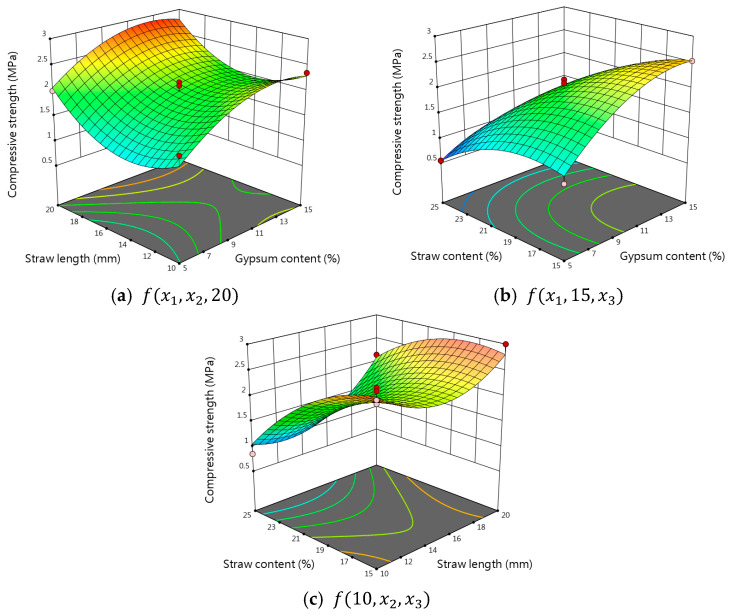
Influence of interaction factors on compressive strength.

**Figure 4:**
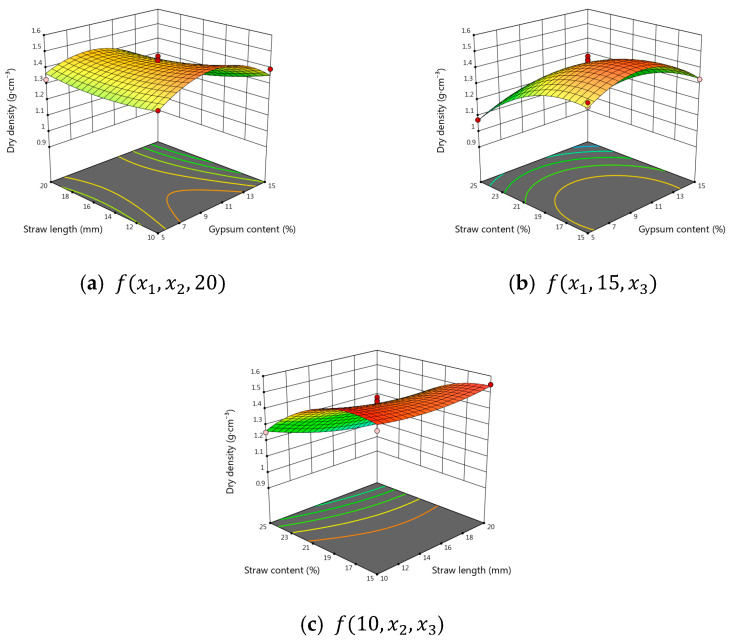
Influence of interaction factors on dry density.

**Figure 5:**
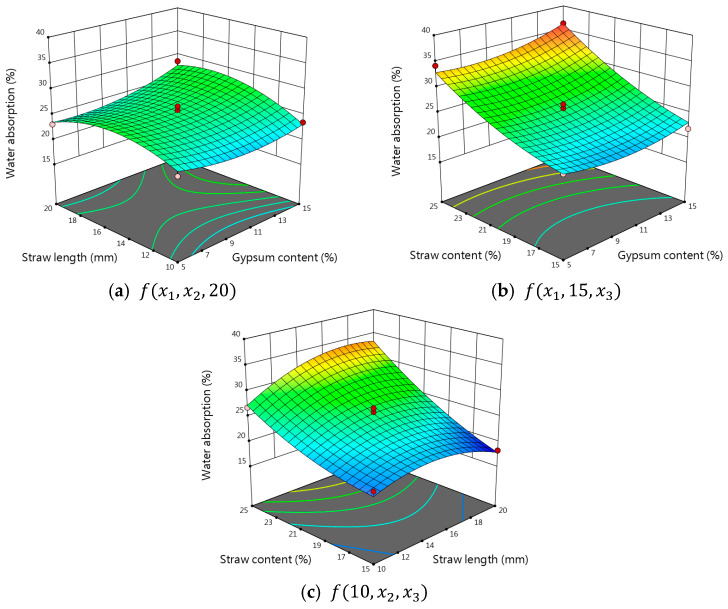
Influence of interaction factors on water absorption rate.

Based on the results of Table 5 and Table 6, interaction effects of independent variables on all responses of composite materials generated by Design Expert software are shown in Figure 3, Figure 4 and Figure 5.

It could be seen from Figure 3 that, when the gypsum content was held constant, the compressive strength initially decreased slightly with increasing straw length, reached a minimum value, and then increased, showing a distinct trough. When the straw length was held constant, the compressive strength first increased rapidly with increasing gypsum content and then decreased slightly, but overall showed an upward trend with increasing gypsum content. Increasing the gypsum content or decreasing the straw content both improve the compressive strength of the composite material. When the gypsum content is below 10%, the compressive strength decreases rapidly with increasing straw content. When the straw content is around 20%, the rate of decrease in compressive strength slows somewhat, but the overall trend remains downward. When the gypsum content was controlled between 5% and 10% and the straw content was between 20% and 25%, the compressive strength reached a minimum value of 0.55 MPa.

Under the same fiber and cement contents, longer palm fibers were more effective than shorter fibers in improving the compressive strength of cement-treated sand. This was mainly attributed to the greater embedded length of the longer fibers, which provided more effective mechanical anchorage and reduced the likelihood of fiber pullout, thereby resulting in higher peak compressive strength [34]. Zeng et al. [35] found that for 100 mm × 100 mm × 100 mm cubic rape straw fiber concrete specimens with a fiber volume fraction of 0.1%, compressive strength rose steadily throughout the process of fiber length increasing from 10–20 mm to 20–30 mm and 30–40 mm. The compressive strength of the composite materials decreased with the increase of straw content addition [36]. These were consistent with the results of this study. This can be attributed to the high interfacial bonding strength between the fibers and the gypsum-based matrix, as the gypsum matrix tends to roughen the fiber surface, thereby enhancing the interfacial adhesion [37]. Meanwhile, the expansion generated during gypsum hydration and hardening further strengthened the interfacial adhesion between straw and the composite matrix, ultimately improving the overall mechanical performance of the composites. Because the fiber length range of rice straw was smaller than the size of testing block, the rice fiber was used as aggregate component, which could be evenly dispersed in cement paste by fully mixing. With the increase of straw content, the cement paste component played the main mechanical role in the testing block decreased. In addition, the smooth waxy layer on the surface of the straw made the adhesion between the straw and the cement matrix worse, retarding phenomenon appeared after the straw was mixed into the basic matrix which would lead to reduction of strength.

It could be seen from Figure 4 that the dry density first increases and then decreases with increasing gypsum content, and decreases with increasing straw length. Dry density initially increases and then decreases with increasing gypsum content, and decreases with increasing straw content. As straw content and straw length increase, the dry density exhibits a linear decreasing trend. Increasing straw content significantly reduces the dry density of the composite material, while straw length shows little change within this range.

This phenomenon can be explained by the inherent lightweight property of building gypsum. With the increase in straw length and content, the volume fraction of straw within the composite material keeps rising. Due to the low density of the fibers, the dry density of the material exhibits a continuous reduction. This is consistent with the findings of Juárez-Alvarado et al. who reported that porosity increased while density decreased with the increase of fibers in the matrix [38].

It could be seen from Figure 5 that the water absorption rate first increases and then decreases slightly with increasing straw length, showing an overall upward trend. With increasing gypsum content, the variation in the water absorption rate is not significant. As both the gypsum content and the straw content increase, the water absorption rate shows a continuous upward trend. As the length of the straw increases, the water absorption rate first rises and then decreases slightly, showing an overall upward trend; as the straw content increases, the water absorption rate continues to rise.

Migneault et al. [39] found that as the aspect ratio of straw gradually increased from 8.3 to 13.0 and further to 21.3, the water absorption of the composite increased correspondingly. Higher straw fiber content enhances the water absorption rate of mortar concrete [40]. The reason for this phenomenon may be that the water consumption in mixing process of gypsum and cement was greater than theoretical value, and after the excess water evaporated, the water seepage channel was formed inside the composite materials, which increased the water absorption rate. The water absorption of cement-based composites is closely related to that of straw fibers. Unmodified wheat straw fibers exhibit extremely high water absorption due to the abundant hydrophilic hydroxyl groups in their structure [41]. Therefore, increasing the content of straw fibers directly leads to a significant rise in the water absorption of the composite material.

### 3.3. Optimization and Model Validation

The optimized goals are shown in Table 7. The design was optimized based on three target parameters: compressive strength, density, and water absorption. By analyzing the data using Design-Expert software and prioritizing the physical properties in the order of compressive strength > water absorption > density, a material that meets the requirements for practical applications can be obtained. The prioritization of compressive strength, water absorption, and density is respectively based on the requirements of structural safety, durability, and lightweight performance. Compressive strength is regarded as the primary indicator to ensure structural integrity, water absorption is used to evaluate durability-related performance, and density reflects the lightweight characteristics of the material.

According to Design Expert software analysis, the best optimization of materials is shown in Table 8. The composite materials produced with m (cement): m (sand) = 1: 3, 15% gypsum content, 20 mm of straw length, and 15% straw content were chosen to be the best set of condition due to the high compressive strength of 3.06 MPa, low dry density of 1.340 g·cm^−3^, and low water absorption rate of 20.8%.

Under the predicted optimal preparation conditions, the rice straw–mortar composite achieved a compressive strength of 3.06 MPa, which is comparable to the 56-day compressive strengths of 0.44–6.72 MPa reported by Niu et al. [6] for corn straw cement-based composites. Moreover, the optimum StarchCrete formulation reported by Seeponkai et al. [42] exhibited a compressive strength of 2.78 MPa, indicating that the optimum compressive strength achieved in the present study was slightly higher.

## 4. Freeze–Thaw Resistance Test of Rice Straw–Mortar Composites

Northeast China experiences a severe winter climate, where building materials used in residential wall systems are subjected to repeated freeze–thaw cycles during service. Consequently, rice straw mortar composites intended for such applications may experience deterioration in durability under freeze–thaw conditions. Therefore, evaluating their performance and durability under freeze–thaw exposure is essential. In this study, the freeze–thaw test was conducted in accordance with the Chinese standard GB/T 20473–2006 “Dry-mixed thermal insulating composition for buildings” [33] and JGJ/T70-2009 “Standard for test method of performance on building mortar” [32], which specify 15 freeze–thaw cycles for frost resistance evaluation. Specimens subjected to 5 and 10 freeze–thaw cycles were also tested for comparison. The effects of additional freeze–thaw cycles on the long-term durability of the developed composites will be investigated in future studies.

### 4.1. Performance Parameters After Freeze–Thaw Cycles

According to the experimental results in Table 4, the quick-freezing method was selected to simulate the outdoor winter freeze–thaw environment, and the effects of winter rainfall and snowfall erosion as well as frost heave damage on the rice straw–mortar composite materials were discussed. All 17 sets of experimental results after 5, 10, and 15 freeze–thaw cycles were tested in turn, and the physical and mechanical properties of the specimens in Table 4 were used as reference values to calculate the compressive strength loss rate and mass loss rate, respectively.

#### 4.1.1. Mass Loss Rate

**Figure 6:**
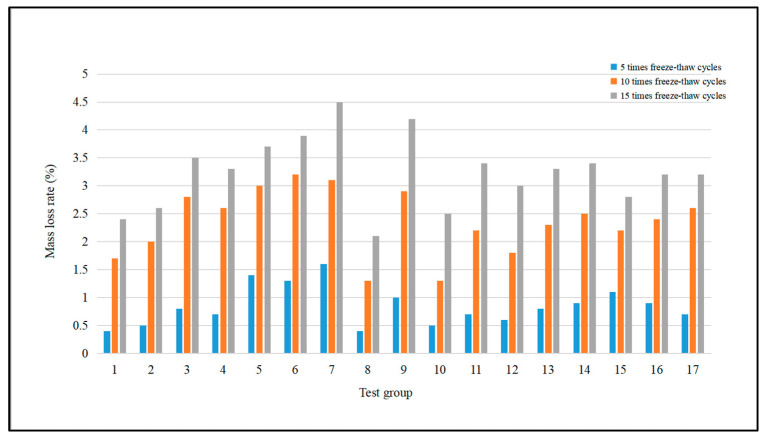
The mass loss rate of testing blocks after Freeze–Thaw Cycles.

After 5, 10, and 15 freeze–thaw cycles, the mass loss rates of 17 sets of specimens were calculated, and the results are shown in Figure 6.

According to the Figure 6, the mass loss rate was positively connected with the number of freeze–thaw cycles, and the mass loss rate of all 17 sets rice straw-mortar composite blocks all increased with the increase of the freeze–thaw cycles. After 5th, 10th, and 15th freeze–thaw cycles, the mass loss rate varied from 0.4% to 1.6%, 1.3% to 3.0%, and 2.1% to 4.5%, respectively. The maximum mass loss rate of the specimens was not higher than 5%, which meant that the composite materials had a certain degree of frost resistance according to the regulation of “Dry-mixed thermal insulating composition for buildings” (GB/T20473-2006) [33].

**Figure 7:**
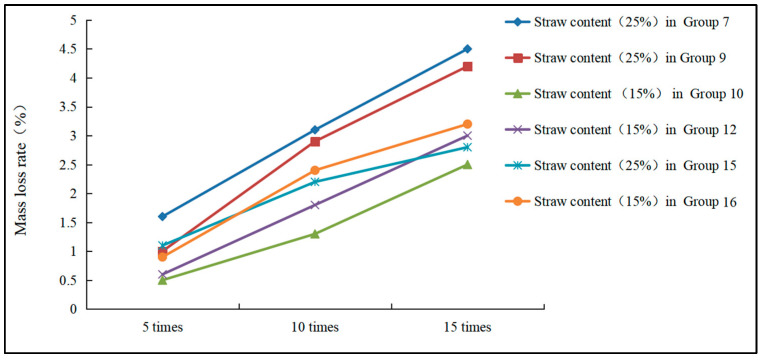
Effect of straw content on mass loss rate under Freeze–Thaw Cycles.

Zhou [43] studied the phosphogypsum-straw composite wall materials, and observed that the straw content had a significant effect on the mass loss rate of mortar samples under the condition of freeze–thaw cycles. Based on previous studies, straw content influences the mass loss rate of composite materials under freeze–thaw conditions. To better reflect the influence of straw content on the mass loss rate of rice straw–mortar composites under freeze–thaw cycles, the experimental results of the 7th and 16th sets, the 9th and 12th sets, and the 10th and 15th sets (as shown in Figure 7) were compared. It was found that composites with higher straw content exhibited greater mass loss after freeze–thaw cycles.

#### 4.1.2. Compressive Strength Loss Rate

**Figure 8:**
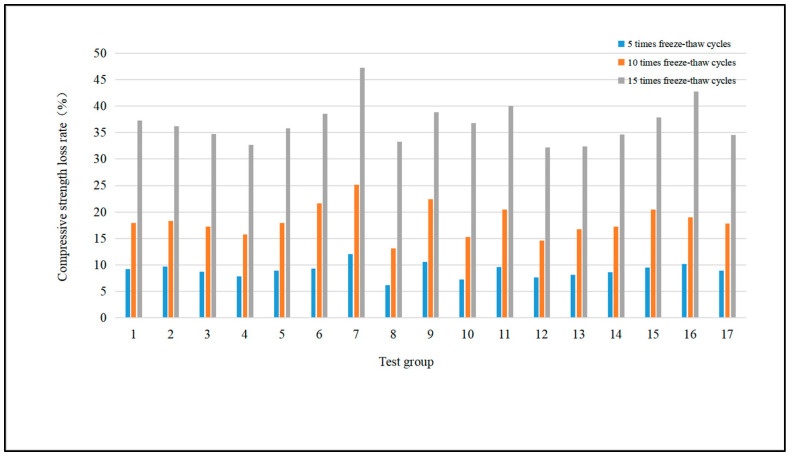
The compressive strength loss rate of testing blocks after Freeze–Thaw Cycles.

After 5, 10 and 15 freeze–thaw cycles, the compressive strength loss rates of 17 sets of specimens were calculated, and the results are shown in Figure 8.

Compressive strength gradually declines, with loss accelerating as the number of freeze–thaw cycles increases [44]. In this study, the compressive strength loss rate of all 17 sets of composite specimens increased with increasing freeze–thaw cycles. This is consistent with the conclusions reported by Li [45]. After 5th, 10th, and 15th freeze–thaw cycles, the compressive strength loss rate varied from 6.2% to 12.1%, 13.11% to 25.2%, and 32.4% to 47.2%, respectively. According to GB/T 20473-2006 “Dry-mixed thermal insulating composition for buildings” [33] test specimens with good frost resistance should exhibit a strength loss rate of no more than 25%. The test specimens exhibited good freeze-thaw resistance after the 5th and 10th freeze-thaw cycles. This may be because, during the initial stages of freeze-thaw, there were certain voids within the straw that could accommodate a certain degree of volume expansion caused by water freezing and the movement of penetrating water. The rate of mass loss and the rate of compressive strength loss of the test specimens remained within the standard range, indicating good freeze-thaw resistance. However, during the 10th to 15th freeze-thaw cycles, sand and gravel began to detach from the surface and edges of the test specimens. Although the mass loss rate remained below 5%, the internal structure became loose at this stage, resulting in a decrease in strength. But the testing block after freeze–thaw cycles still retained a certain strength, which could be applied to non-bearing walls and partitions [46].

**Figure 9:**
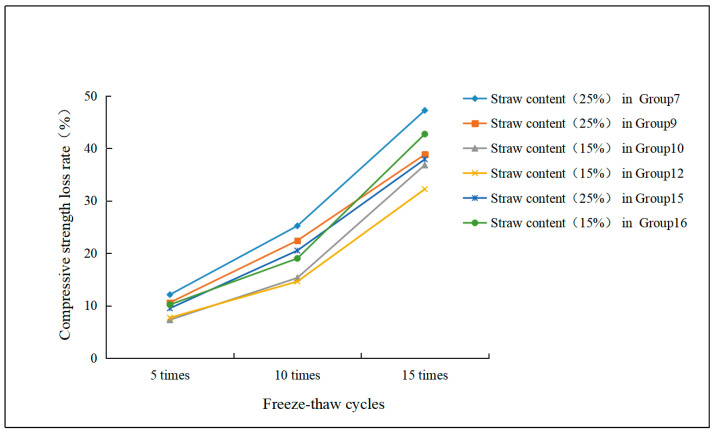
Effect of straw content on compressive strength loss rate under Freeze–Thaw Cycles.

In Zhou’s [43] study on lightweight wall materials made from phosphogypsum and crop straw, the strength loss rate of the test specimens increased as the straw content rose. Li [45] found that in alkali-activated slag-fly ash composite materials containing 4% straw, compressive strength decreased as the number of freeze-thaw cycles increased. Based on previous studies, the straw content also has an influence on the strength loss rate of composite materials under freeze–thaw conditions. To better reflect the impact of straw content on the compressive strength loss rate of rice straw–mortar composites, the results of the 7th and 16th sets, the 9th and 12th sets, and the 10th and 15th sets were compared. It was found that composites with higher straw content exhibited a greater compressive strength loss rate (as shown in Figure 9).

### 4.2. Analysis of Testing Results After 15 Times Freeze–Thaw Cycles

This study primarily analyzes the mass loss rate, strength loss rate, compressive failure mode and water absorption rate of test specimens after 15 freeze-thaw cycles to investigate the effects of freeze-thaw cycles on material properties. Table 9 presents the test data on the mass loss rate, strength loss rate, and water absorption rate of 17 groups of test specimens after 15 freeze-thaw cycles.

#### 4.2.1. Compressive Failure of Testing Block Under Freeze–Thaw Cycles

**Figure 10:**
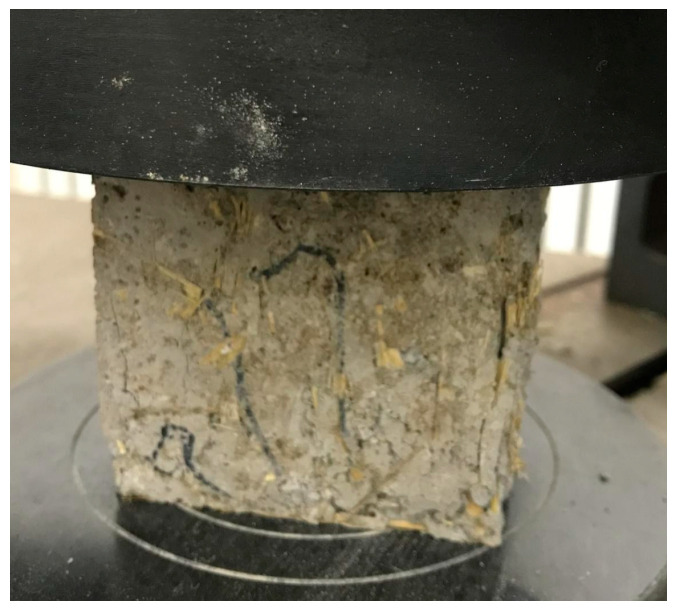
Compression failure of specimens in normal temperature.

**Figure 11:**
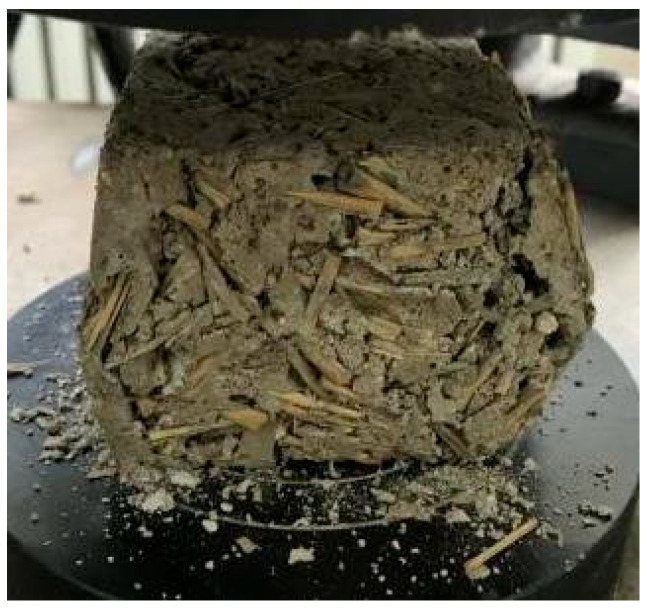
Compression failure of specimens after 15 times Freeze–Thaw Cycles.

After 15 freeze–thaw cycles, distinct vertical cracks appeared on the surface of the specimens as the testing machine began to apply load, accompanied by the detachment of surface aggregates; however, the specimens had not yet reached their ultimate compressive strength at this stage. This may be because, compared to ordinary mortar specimens, the rice straw mortar composite contains uniformly dispersed straw fibers that still provide a certain degree of mechanical support. However, as the load continued to increase, a primary crack penetrated the entire specimen, and the test was terminated. Referring to Figure 10 and Figure 11, when the ultimate load was reached, no obvious cracks appeared on the surface of the specimens at room temperature. However, after 15 freeze-thaw cycles, the specimen surfaces exhibited distinct vertical cracks and significant loss of aggregate, and the specimens no longer retained their original cubic shape. Under freeze-thaw cycles, the strength of most composite materials deteriorates and the degree of damage increases [47].

#### 4.2.2. Water Absorption Rate After Freeze–Thaw Cycles

Water absorption rate affected the durability of materials, and partly reflected the compactness and strength of materials. Ishigami [48] found that freeze-thaw damage significantly increases the water absorption rate and water absorption capacity of concrete. Qi et al. [49] obtained that the internal structure of the aggregate damaged due to the increase of frost heave and pore volume when lightweight aggregates with high moisture content subjected to freeze–thaw cycles action. These were consistent with the results which could be gotten from the Figure 10 and Figure 11. The tightness of material’s internal connection played a decisive role in the compressive strength. If the water absorption rate was large, the internal connection was relatively loose, and the mechanical properties of the material would reduce. According to the testing results of Table 9, the water absorption rate of most testing blocks generally increased after 15 freeze–thaw cycles under room temperature conditions. The reason may be that, as the internal temperature of the freezer decreased, the temperature inside the specimens also continued to decrease. The straw structure contained a large amount of unfrozen water, which expanded upon freezing and generated internal pressure. When the solution in the rice straw–mortar composites thawed, the volume expansion caused by ice formation remained unchanged, and the newly formed voids were refilled with water when the specimens were soaked again. After repeated freeze–thaw cycles, the water absorption rate increased. Therefore, in some special environmental conditions, waterproof measures must be adopted to reduce the damage of freeze–thaw cycles to the strength and structural stability of rice straw-mortar composite materials.

#### 4.2.3. Microstructural Evolution During Freeze–Thaw Cycles

**Figure 12:**
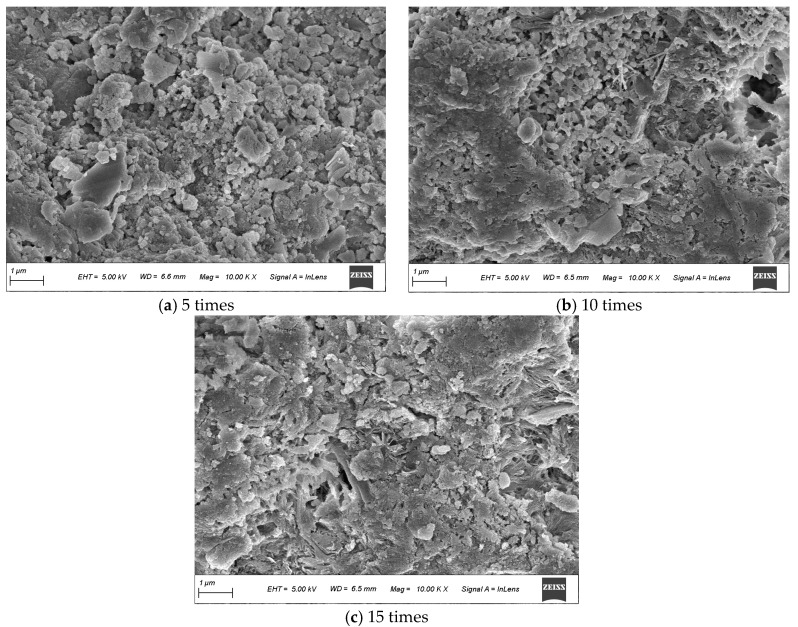
Microstructural images after freeze–thaw cycles.

As shown in Figure 12, the first specimen showed a relatively dense matrix with limited pore development, where rice straw porosity provided some resistance to early freeze–thaw damage and the internal skeleton remained intact. In the second specimen, increased voids and interfacial gaps indicated the onset of matrix loosening. In the third specimen, extensive microcracks, pore growth, and significant debonding of hydration products were observed, reflecting severe degradation of the three-phase structure. Repeated freeze–thaw cycles promoted water accumulation and progressive internal damage, weakening the load-bearing skeleton. Under loading, stress concentration in damaged zones led to crack propagation and eventual formation of a dominant through-crack. The fiber–matrix interface was irreversibly degraded, and fibers provided only limited bridging capacity. Ultimately, freeze–thaw action resulted in microstructural breakdown, manifested macroscopically as strength loss and surface spalling.

## 5. Conclusions

In this study, the response surface test was designed through Design-Expert software to analyze the physical and mechanical properties and freeze–thaw resistant performance of the composite materials with different gypsum content, straw length, and straw content. The main conclusions are as follows:

(1) The straw content had a significant influence on the compressive strength, dry density, and water absorption rate. With increasing straw content, the compressive strength and dry density of the rice straw–mortar composites decreased continuously, while the water absorption rate increased.
(2) The optimal mix proportion of the rice straw–mortar composite was obtained at a cement-to-sand mass ratio of 1:3, a gypsum content of 15%, a rice straw length of 20 mm, and a rice straw content of 15%. Under these conditions, the composite achieved a compressive strength of 3.06 MPa, a density of 1.340 g·cm^−3^, and a water absorption rate of 20.8%. The calculated results indicate that the compressive strength of the rice straw mortar composite meets the requirements of M2.5 grade mortar. Based on these results, the material shows potential for use in non-load-bearing wall applications.
(3) The compressive strength loss rate and mass loss rate increased with increasing freeze–thaw cycles and straw content. Compared with the conditions at room temperature, the water absorption of the vast majority of specimens generally increased after 15 freeze–thaw cycles. The compressive strength loss rate of the rice straw–mortar composites ranged from 32.2% to 47.2%, the mass loss rate ranged from 2.1% to 4.5%, and the water absorption rate ranged from 21.8% to 37.2%. Although the specimens exhibited a compressive strength loss of more than 25%, the rice straw–mortar composite remained capable of retaining sufficient residual strength after freeze–thaw treatment, indicating its suitability for non-load-bearing wall applications.

The optimized mixture identified in this study provides guidance for the efficient utilization of straw resources in cement-based composites. The identified optimal combination demonstrates the feasibility of incorporating straw into cement-based systems while maintaining satisfactory performance, thereby improving resource utilization efficiency and reducing environmental burden. Overall, this work supports the efficient utilization of straw resources and provides a reference for the development of more sustainable cementitious materials.

## Figures and Tables

**Table 1:** The physical and mechanical properties of cement.

Specific Surface Area	Density	Setting Time (min)		Compressive Strength (MPa)	
(cm^2^·g^−1^)	(g·cm^−3^)	Initial Setting Time	Final Setting Time	3d	28d
3245	3.15	183	237	25.4	49.5

**Table 2:** Gypsum specification.

Technical Specification		Values
Strength (MPa)	Flexural strength	≥1.8
	Compressive strength	≥2.9
Fineness (%)	0.2 mm square sieve residue	≤15.0
Coagulation time (min)	Initial setting time ≥, Final setting time ≤	6, 30

**Table 3:** Coded levels for independent variables used in the experiment.

Factors Levels	$x_{1}$ (%)	$x_{2}$ (mm)	$x_{3}$ (%)
1	15	20	25
0	10	15	20
−1	5	10	15
$\Delta_{j}$	5	5	5

Where, $x_{1}$ is gypsum content (%); $x_{2}$ is straw length (mm); $x_{3}$ is straw content (%).

**Table 4:** Physical and mechanical properties of composite materials based on the Box-Behnken experimental design.

Number	$x_{1}$ (%)	$x_{2}$ (mm)	$x_{3}$ (%)	$y_{f}$ (MPa)	Residual	$y_{\rho }$ (g·cm^−3^)	Residual	$y_{w}$ (%)	Residual
1	15	20	20	2.60	−0.1912	1.206	−0.0041	28.6	0.8875
2	15	10	20	2.35	0.0588	1.394	0.0384	23.5	0.3875
3	10	15	20	1.90	−0.0980	1.432	−0.0024	25.3	−0.3200
4	10	15	20	2.17	0.1720	1.473	0.0386	24.9	−0.7200
5	5	10	20	1.65	0.1913	1.375	0.0041	22.6	−0.8875
6	5	20	20	2.00	−0.0587	1.329	−0.0384	23.1	−0.3875
7	5	15	25	0.55	0.0050	1.075	0.0042	34.2	1.30
8	10	20	15	3.00	0.1963	1.549	0.0084	18.2	0.4125
9	10	10	25	0.85	−0.1962	1.256	−0.0084	26.8	−0.4125
10	15	15	15	2.53	−0.0050	1.331	−0.0043	21.8	−1.30
11	10	20	25	2.15	0.0538	1.130	0.0341	32.4	−0.9125
12	10	10	15	2.70	−0.0537	1.487	−0.0341	20.2	0.9125
13	10	15	20	2.10	0.1020	1.452	0.0176	25.9	0.2800
14	10	15	20	1.85	−0.1480	1.446	0.0116	26.7	1.08
15	15	15	25	0.95	0.1375	0.921	−0.0300	36.3	0.0250
16	5	15	15	1.10	−0.1375	1.418	0.0300	22.6	−0.0250
17	10	15	20	1.97	−0.0280	1.369	−0.065	25.3	−0.320

Where, $x_{1}$ is gypsum content (%); $x_{2}$ is straw length (mm); $x_{3}$ is straw content (%); $y_{f}$ is compressive strength in normal temperature condition (MPa); $y_{\rho }$ is dry density in normal temperature condition (g·cm^−3^); $y_{w}$ is water absorption rate in normal temperature condition (%).

**Table 5:** Regression equations for physical and mechanical properties of rice straw-mortar composite materials.

Physical and Mechanical Properties	Response Surface Model	$R_{\mathrm{adj}}^{2}$	Predicted R^2^	Adeq Precision	C.V.%
Compressive strength	$\begin{array}{c} y_{f}=1.29075+0.59545x_{1}-0.7617x_{2}+0.38465x_{3}-0.0103x_{1}x_{3}+0.01x_{2}x_{3}\\ -0.01481x_{1}^{2}+0.02089x_{2}^{2}-0.01381x_{3}^{2} \end{array}$	0.9186	0.5661	14.9127	10.36
Dry density	$\begin{array}{c} y_{\rho }=-0.607775+0.137135x_{1}+0.00784x_{2}+0.174745x_{3}\\ -0.005553x_{1}^{2}-0.004373x_{3}^{2} \end{array}$	0.9299	0.7132	17.5046	3.30
Water absorption rate	$\begin{array}{c} y_{w}=38.3175-2.3395x_{1}+1.547x_{2}-2.7015x_{3}+0.076x_{2}x_{3}\\ +0.0631x_{1}^{2}-0.1099x_{2}^{2}+0.0611x_{3}^{2} \end{array}$	0.9435	0.6807	20.9913	4.45

Where, $x_{1}$ is gypsum content (%); $x_{2}$ is straw length (mm); $x_{3}$ is straw content (%); $y_{f}$ is compressive strength in normal temperature condition (MPa); $y_{\rho }$ is dry density in normal temperature condition (g·cm^−3^); $y_{w}$ is water absorption rate in normal temperature condition (%).

**Table 6:** Coefficient values and variance analysis of the fitting model for different responses of composite materials.

Response	Source	*df*	Sum of Squares	Mean Squares	*F* Value	*p* Value	Significance
Compressive strength	Model	9	7.39	0.82	21.05	0.0003	**
	$x_{1}$	1	1.22	1.22	31.40	0.0008	**
	$x_{2}$	1	0.60	0.60	15.51	0.0056	**
	$x_{3}$	1	2.92	2.92	74.77	<0.0001	**
	$x_{1}x_{2}$	1	2.5 × 10^−3^	2.5 × 10^−3^	0.064	0.8074	-
	$x_{1}x_{3}$	1	0.27	0.27	6.80	0.0350	*
	$x_{2}x_{3}$	1	0.25	0.25	6.41	0.0391	*
	$x_{1}^{2}$	1	0.58	0.58	14.80	0.0063	**
	$x_{2}^{2}$	1	1.15	1.15	29.45	0.0010	**
	$x_{3}^{2}$	1	0.50	0.50	12.87	0.0089	**
	Residual	7	0.27	0.039			
	Lack of fit	3	0.20	0.067	3.70	0.1192	
	Pure Error	4	0.072	0.018			
	Total Error	16	7.66				
Dry density	Model	9	0.4268	0.0474	24.58	0.0002	**
	$x_{1}$	1	0.0149	0.0149	7.71	0.0274	*
	$x_{2}$	1	0.0111	0.0111	5.75	0.0475	*
	$x_{3}$	1	0.2461	0.2461	127.56	<0.0001	**
	$x_{1}x_{2}$	1	0.0050	0.0050	2.61	0.15	-
	$x_{1}x_{3}$	1	0.0011	0.0011	0.58	0.4705	-
	$x_{2}x_{3}$	1	0.0088	0.0088	4.58	0.0696	-
	$x_{1}^{2}$	1	0.0811	0.0811	42.07	0.0003	**
	$x_{2}^{2}$	1	0.0039	0.0039	2.02	0.1982	-
	$x_{3}^{2}$	1	0.0503	0.0503	26.09	0.0014	**
	Residual	7	0.0135	0.0019			
	Lack of fit	3	0.0073	0.0024	1.56	0.33	-
	Pure Error	4	0.0062	0.0016			
	Total Error	16	0.4403				
Water absorption rate	Model	9	364.29	40.48	30.7	<0.0001	**
	$x_{1}$	1	7.41	7.41	5.62	0.0496	*
	$x_{2}$	1	10.58	10.58	8.02	0.0253	*
	$x_{3}$	1	274.95	274.95	208.51	<0.0001	**
	$x_{1}x_{2}$	1	5.29	5.29	4.01	0.0853	-
	$x_{1}x_{3}$	1	2.1	2.1	1.59	0.2471	-
	$x_{2}x_{3}$	1	14.44	14.44	10.95	0.0130	*
	$x_{1}^{2}$	1	10.48	10.48	7.95	0.0258	*
	$x_{2}^{2}$	1	31.78	31.78	24.10	0.0017	**
	$x_{3}^{2}$	1	9.82	9.82	7.45	0.0294	*
	Residual	7	9.23	1.32			
	Lack of fit	3	7.26	2.42	4.92	0.0789	-
	Pure Error	4	1.97	0.49			
	Total Error	16	373.52				

Where, $x_{1}$ is gypsum content (%); $x_{2}$ is straw length (mm); $x_{3}$ is straw content (%); “**” means extremely significant difference at *p* < 0.01 level; “*” means generally significant difference at *p* < 0.05 level.

**Table 7:** Optimization goal of variables during the experimental production rice straw-mortar composite materials.

Variable	Goal	Level of Importance
Independent		
Gypsum content (%)	In range (5–15)	
Straw length (mm)	In range (10–20)	
Straw content (%)	In range (15–25)	
Response		
Compressive strength (MPa)	Maximize	First
Dry density (g·cm^−3^)	Minimize	Third
Water absorption rate (%)	Minimize	Second

**Table 8:** Optimum conditions for producing rice straw-mortar composite materials.

Gypsum Content (%)	Straw Length (mm)	Straw Content (%)	Compressive Strength (MPa)	Dry Density (g·cm^−3^)	Water Absorption Rate (%)
15	20	15	3.06	1.340	20.8

**Table 9:** The performance change of composite materials after 15 freeze–thaw cycles.

Number	Compressive Strength(MPa)		Strength Loss Rate	Quality Loss Rate	Absorption Rate(%)	
	Comparison Test Block	Freeze-Thaw Test Block			Comparison Test Block	Freeze-Thaw Test Block
1	2.60	1.63	37.3	2.4	28.6	33.2
2	2.35	1.50	36.2	2.6	23.5	27.9
3	1.90	1.25	34.7	3.5	25.3	23.4
4	2.17	1.46	32.7	3.3	24.9	28.3
5	1.65	1.06	35.8	3.7	22.6	27.1
6	2.00	1.23	38.5	3.9	23.1	21.8
7	0.55	0.29	47.2	4.5	34.2	30.5
8	3.00	2.00	33.3	2.1	18.2	22.6
9	0.85	0.52	38.8	4.2	26.8	24.7
10	2.53	1.60	36.8	2.5	21.8	26.0
11	2.15	1.29	40.0	3.4	32.4	36.8
12	2.70	1.83	32.2	3.0	20.2	25.3
13	2.10	1.42	32.4	3.3	25.9	28.8
14	1.85	1.21	34.6	3.4	26.7	27.1
15	0.95	0.59	37.9	2.8	36.3	37.2
16	1.10	0.63	42.7	3.2	22.6	25.9
17	1.97	1.29	34.5	3.2	25.3	28.6

## Data Availability

The original contributions presented in this study are included in the article. Further inquiries can be directed to the corresponding author.
